# Clinical stability after compassionate use of intranasal esketamine in treatment-resistant depression

**DOI:** 10.1192/j.eurpsy.2022.762

**Published:** 2022-09-01

**Authors:** D. Hernandez Huerta, A. De Santiago Diaz, C. Rodriguez Gomez-Carreño, M.A. Abril Garcia, F. Toledo Romero, A. Guerrero Morcillo, C.J. Martinez Pastor, M. Vega Piñero

**Affiliations:** 1 Ramon y Cajal University Hospital, Department Of Psychiatry, Madrid, Spain; 2 University Hospital Marqués de Valdecilla, Department Of Psychiatry, Santander, Spain; 3 University Hospital of Ciudad Real, Department Of Psychiatry, Ciudad Real, Spain; 4 University Hospital Gómez Ulla, Department Of Psychiatry, Madrid, Spain; 5 University Hospital Virgen de la Arrixaca, Department Of Psychiatry, Murcia, Spain; 6 General Hospital of Villarrobledo, Department Of Psychiatry, Albacete, Spain; 7 University Hospital of Elche, Department Of Psychiatry, Elche, Spain; 8 University Hospital Ramón y Cajal, Department Of Psychiatry, Madrid, Spain

**Keywords:** Depression, esketamine, Treatment-resistant depression

## Abstract

**Introduction:**

The compassionate use of intranasal esketamine is approved in Spain for treatment-resistant depression (TRD).

**Objectives:**

The objective of the study is to assess the clinical stability in the medium-term follow-up of patients with TRD after esketamine use.

**Methods:**

Descriptive, retrospective and multicenter study carried out in Spain. Patients with TRD who had received esketamine treatment, and for whom there were clinical data of subsequent evolution, were included. The scores on the MADRS and Hamilton scales were changed into scores on the CGI scale according to the studies by Leucht et al. The Student’s t test was performed to assess differences in the CGI.

**Results:**

Eleven patients were included: 72.7% were women and the mean age was 56 (SD: 12.9). The maximum dose of esketamine used was 84mg in 63.7%. The onset of antidepressant action was observed from the 1st dose in 72.6% of the patients. The mean time in treatment was 6.6 months (SD: 2.3) and 90.9% reached remission criteria. After 7.4 months (SD: 3.0) from the end of the treatment, 90.9% remained in remission and without visits to the emergency room or hospitalization for psychiatric reasons. The mean baseline score on the CGI-SI was 5.7 points, at the end of the treatment was 1.2 points and after longitudinal follow-up it was 1. Statistically significant differences were observed (p<0.001) both at the end of the treatment and in the post-esketamine follow-up compared with baseline score.

**Conclusions:**

In our sample, the use of esketamine in TRD shows clinical stability in the medium-term follow-up.

**Disclosure:**

Daniel Hernández has participated in medical meetings and/or received payment for presentations from Otsuka, Lundbeck, Janssen, Angelini, Casen Recordati, and Ferrer.

